# Upregulated lncRNA-UCA1 contributes to progression of hepatocellular carcinoma through inhibition of miR-216b and activation of FGFR1/ERK signaling pathway

**DOI:** 10.18632/oncotarget.3219

**Published:** 2015-02-11

**Authors:** Feng Wang, Hou-Qun Ying, Bang-Shun He, Yu-Qin Pan, Qi-Wen Deng, Hui-Ling Sun, Jie Chen, Xian Liu, Shu-Kui Wang

**Affiliations:** ^1^ Central Laboratory, Nanjing First Hospital, Nanjing Medical University, Nanjing, Jiangsu, China; ^2^ Medical college, Southeast University, Nanjing, Jiangsu, China; ^3^ Department of Life Sciences, Nanjing Normal University, Nanjing, Jiangsu, China

**Keywords:** hepatocellular carcinoma, lncRNA, UCA1, miR-216b, FGFR1

## Abstract

The long non-coding RNA (lncRNA) urothelial carcinoma-associated 1 (UCA1) has been recently shown to be dysregulated, which plays an important role in the progression of several cancers. However, the biological role and clinical significance of UCA1 in the carcinogenesis of hepatocellular carcinoma (HCC) remain unclear. Herein, we found that UCA1 was aberrantly upregulated in HCC tissues and associated with TNM stage, metastasis and postoperative survival. UCA1 depletion inhibited the growth and metastasis of HCC cell lines *in vitro* and *in vivo*. Furthermore, UCA1 could act as an endogenous sponge by directly binding to miR-216b and downregulation miR-216b expression. In addition, UCA1 could reverse the inhibitory effect of miR-216b on the growth and metastasis of HCC cells, which might be involved in the derepression of fibroblast growth factor receptor 1 (FGFR1) expression, a target gene of miR-216b, and the activation of ERK signaling pathway. Taken together, our data highlights the pivotal role of UCA1 in the tumorigenesis of HCC. Moreover, the present study elucidates a novel lncRNA- miRNA-mRNA regulatory network that is UCA1-miR-216b-FGFR1-ERK signaling pathway in HCC, which may help to lead a better understanding the pathogenesis of HCC and probe the feasibility of lncRNA-directed diagnosis and therapy for this deadly disease.

## INTRODUCTION

Hepatocellular carcinoma (HCC) ranks the sixth most common tumors and the third leading cause of cancer-related mortality worldwide. Although the majority of the cases occur in Asia and Africa, the incidence of HCC in the United States has been rising over the past three decades and currently represents the fastest growing cause of cancer-related deaths among men [1]. Moreover, epidemiologic evidence demonstrates that the medical and economic burden of HCC will still soar drastically in Western populations during the next decade [2]. Despite scientific efforts and significant progress in understanding the basic cellular event in HCC, the precise mechanisms underlying liver carcinogenesis are still unknown and 5-year survival rates have not changed much during the past several years. Therefore, novel diagnostic biomarkers and therapeutic strategies are urgently needed in order to improve the prognosis of patients with HCC [3, 4].

Generally, non-coding RNAs (ncRNAs) are loosely grouped into two major classes based on transcript size: small ncRNAs and long non-coding RNAs (lncRNAs). MicroRNAs (miRNAs) are a class of small ncRNAs that bind to the 3′-untranslated region (3′-UTR) of mRNAs, thereby inhibiting mRNAs translation or promoting mRNAs degradation. Mounting evidence has showed that miRNAs play a central role in the regulation of cell development, differentiation, proliferation and apoptosis. On the other hand, the recent discovery of lncRNAs and the elucidation of their functions have disclosed a new layer of complexity underlying the regulation of gene expression in cancer. Documents have demonstrated that several lncRNAs, such as lncRNA HULC, RERT and HOTTIP/HOXA13, etc., are dysregulated in HCC and closely related to tumorigenesis, metastasis, prognosis, diagnosis, and drug resistance [5–7], opening up a new avenue for investigating the occurrence and development molecular mechanisms of HCC.

LncRNAs are functionally very diverse, which can act as molecular signals, tethers, decoys, guides and/or scaffolds at nearly every level of gene regulation: epigenetic, transcriptional, posttranscriptional, and translational [8, 9]. Recently, studies have showed that lncRNAs may act as competing endogenous RNAs (ceRNAs), namely miRNA sponges or antagomirs, which may downregulate miRNAs expression and activities, subsequently modulating the derepression of miRNA targets at the level of post-transcriptional regulation [10–12]. It has been showed that thousands of lncRNAs possess cell type-, tissue type-, developmental stage- and disease-specific expression patterns and localization, suggesting that individual lncRNA may be potent natural miRNA sponges in certain conditions [13]. Yet, it is not fully clear whether this unique function of lncRNAs is involved in the carcinogenesis of HCC.

Human urothelial carcinoma associated 1 (UCA1) gene is located in chromosome 19p13.12, which has three exons and encodes two transcripts. LncRNA UCA1 has two isoforms: one is 1.4 kb in length [14]; another isoform is 2.2 kb in length, which has also been identified by a different group as cancer upregulated drug resistant (CUDR) [15]. Several groups have reported that UCA1 is highly expressed in bladder cancer, breast cancer, colorectal cancer, etc. [14, 16, 17], suggesting that UCA1 may serve as a biomarker for the diagnosis of these cancers. Moreover, UCA1 enhances bladder cancer cell proliferation and metastasis through PI3K, Wnt or Akt signaling pathway [18–20]. In addition, miR-1 plays a tumor suppressive role via downregulating UCA1 in bladder cancer and UCA1 participates in cancer cell glucose metabolism through the cascade of mTOR-STAT3/miR143-hexokinase 2 (HK2) [21–22], indicating a positive interaction between UCA1 and miRNAs in cancer cells. Nevertheless, up to now, there is no relevant report about the relationship between UCA1 and the progression of HCC. Thus, the role of UCA1 in HCC and its underlying mechanism remain to be determined.

In the present study, we show that UCA1 is overexpressed in HCC and it may play an oncogenic role in promoting malignancy of HCC cells, including proliferation and metastasis *in vitro* and *in vivo*. Importantly, mechanistic analysis reveals that UCA1 may function as an endogenous sponge to upregulate the expression of fibroblast growth factor receptor 1 (FGFR1) through directly binding and inhibiting the expression of miR-216b, which is also involved in the activation of ERK signaling pathway. Our present results provide the first evidence for a novel lncRNA-miRNA -mRNA regulatory network that is UCA1- miR-216b-FGFR1-ERK signaling pathway in HCC, shedding new light on the diagnosis and treatment for this deadly disease.

## RESULTS

### UCA1 is aberrantly upregulated in HCC tissues and associated with disease progression

**Figure 1:**
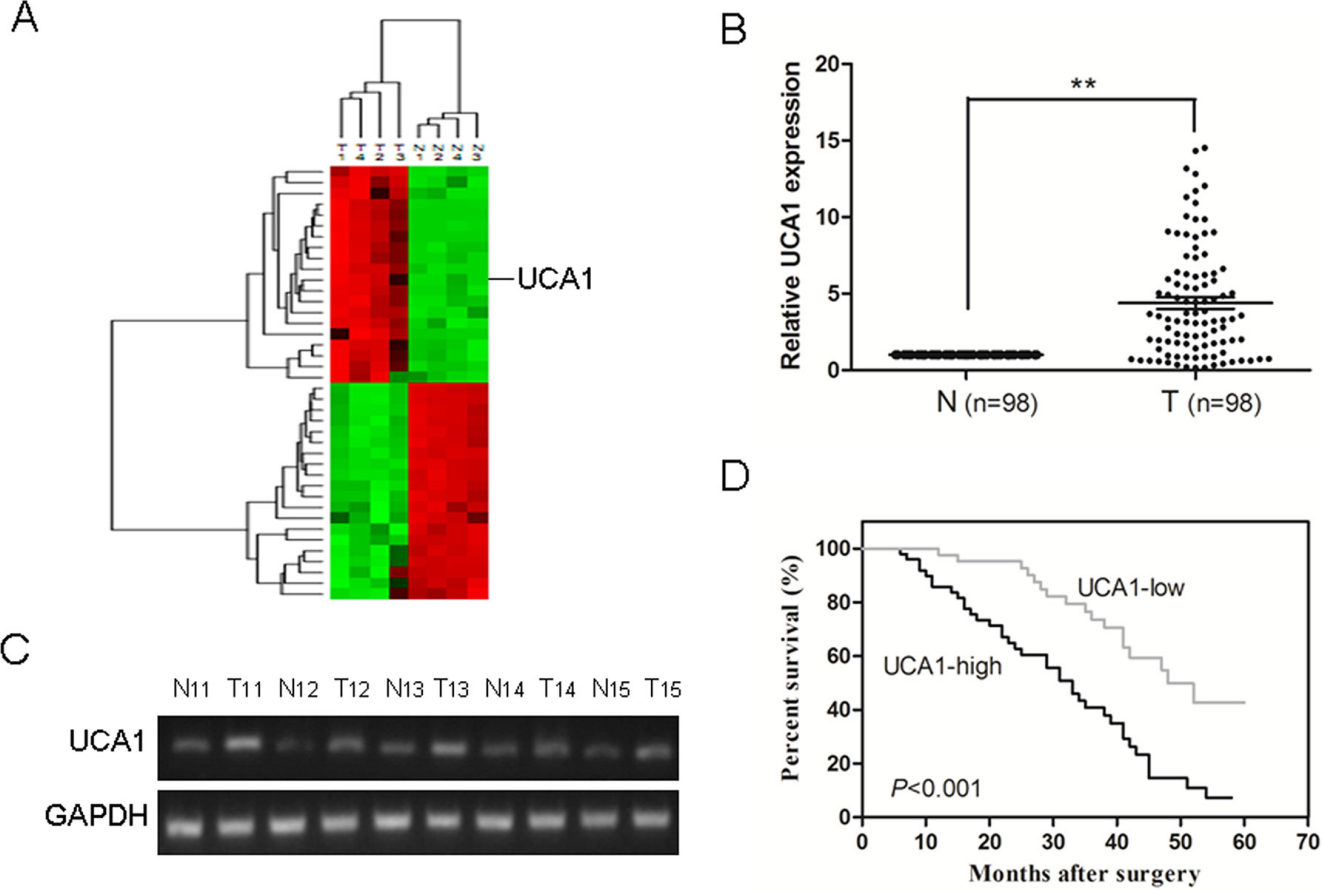
Relative UCA1 expression in HCC tissues and its relationship with overall survival of HCC patients **(A)** Unsupervised hierarchical clustering analysis on the most 20 significantly dysregulated lncRNAs resulted from microarray assay. The normalized expression values are represented in shades of red and green, indicating expression above and below the median expression value across all of the samples. **(B)** UCA1 expression was examined by qRT-PCR and normalized to GAPDH expression in 98 pairs of HCC tissues (T) compared with corresponding nontumourous liver specimens (N), **P < 0.001. **(C)** Semiquantitative RT-PCR analysis of UCA1 expression from 5 patients with HCC; T, tumor tissues; N, corresponding adjacent normal tissues. **(D)** Kaplan-Meier survival curve and log-rank test were used to evaluate whether UCA1 expression level was associated with overall survival rate. Patients were segregated into UCA1-high group and UCA1-low according to the median of UCA1 expression in HCC.

Firstly, the Agilent G3 Human GE Microarray (8 × 60 K) was used to analyze lncRNA expression profiles in 4 HCC tissues and paired corresponding nontumourous tissues. Fold change greater than 2 and *P* value less than 0.05 between tumor tissues and adjacent normal tissues were set as the criteria in filtering differently expressed lncRNAs. Results of unsupervised hierarchical clustering analysis on the top 20 significantly dysregulated lncRNAs were shown in Figure 1A. We further analyzed the expression of the top 20 upregulated lncRNAs in HCC tissues by qRT-PCR and finally focused on UCA1 in our study.

Then, qRT-PCR analysis was performed to determine the expression level of UCA1 in 98 pairs of human primary HCC and corresponding nontumourous liver specimens. We found that the expression of UCA1 in HCC tissues was conspicuously higher than that observed in pair-matched adjacent nontumourous tissues, (*P* < 0.001, Figure 1B). The electrophoretogram of RT-PCR products further confirmed that UCA1 was over-expressed in HCC tissues (Figure 1C). Clinicopathological analysis showed that UCA1 was significantly correlated with advanced TNM stage (*P* < 0.001) and metastasis (*P* < 0.001); whereas, there was no significant correlation between UCA1 and other clinicopathological characteristics such as gender, age, tumor size, serum AFP level and degree of histological differentiation, (*P* > 0.05, Table 1). In addition, to understand the prognostic significance of UCA1 upregulation in HCC, we analyzed the relationship between UCA1 expression in HCC and patient survival and found that high UCA1 expression was significantly associated with a poor 5-year overall survival rate in our HCC cohort, (*P* < 0.001, Figure 1D). Univariate and multivariate Cox proportional hazards analyses showed that UCA1, as well as TNM stage and metastasis, were identified to be independent prognostic factors for survival in HCC patients (Table 2). Collectively, these results suggest that the upregulation of UCA1 may be involved in development, progression and prognosis of the majority of human HCC.

**Table 1:** Correlation between clinicopathological characteristics and UCA1 expression levels in HCC patients

Characteristics	Number of patients	Low UCA1expression (%)	High UCA1expression (%)	*P* value
Gender				0.233
Male	85	40 (81.6)	45 (91.8)	
Female	13	9 (18.4)	4 (8.2)	
Age (years)				0.409
< 55	59	32 (65.3)	27 (55.1)	
≥ 55	39	17 (34.7)	22 (44.9)	
Tumor size (cm)				0.068
< 5	46	28 (57.1)	18 (36.7)	
≥ 5	52	21 (42.9)	31 (63.3)	
Serum AFP (ng/mL)				0.117
< 20	28	18 (36.7)	10 (20.4)	
≥ 20	70	31 (63.3)	39 (79.6)	
HBsAg				0.268
Negative	8	6 (12.2)	2 (4.1)	
Positive	90	43 (87.8)	47 (95.9)	
Liver cirrhosis				0.307
Absence	19	12 (24.5)	7 (14.3)	
Presence	79	37 (75.5)	42 (85.7)	
Histological differentiation				0.106
Well	19	13 (26.5)	6 (12.2)	
Moderate	34	18 (36.7)	16 (32.7)	
Poor	45	18 (36.7)	27 (55.1)	
TNM stage				< 0.001
I + II	43	31 (63.3)	12 (24.5)	
III + IV	55	18 (36.7)	37 (75.5)	
Metastasis				< 0.001
No	57	38 (77.6)	19 (38.8)	
Yes	41	11 (22.4)	30 (61.2)	

**Table 2:** Univariate and multivariate regression analyses of parameters associated with prognosis of HCC patients

Characteristics	Subset	Univariate analysis		Multivariate analysis	
		Hazard ratio (95% CI)	*P* value	Hazard ratio (95% CI)	*P* value
Gender	Male/Female	1.153 (0.670–1.983)	0.727	-	-
Age	< 55/≥ 55	1.220 (0.710–2.096)	0.471	-	-
Tumor size (cm)	< 5/≥ 5	1.735 (1.011–2.976)	0.045	1.514 (0.881–2.602)	0.133
Serum AFP (ng/mL)	< 20/≥ 20	1.767 (1.022–3.055)	0.027	1.402 (0.816–2.408)	0.221
HBV infection	Positive/Negative	1.308 (0.761–2.247)	0.332	-	-
Liver cirrhosis	Presence/Absence	1.504 (0.876–2.582)	0.139	-	-
Histological differentiation	Well + Moderate/Poor	1.618 (0.943–2.777)	0.081	-	-
TNM stage	I + II/III + IV	2.758 (1.599–4.757)	< 0.001	2.020 (1.169–3.488)	0.012
Metastasis	Yes/No	3.206 (1.858–5.530)	< 0.001	2.332 (1.351–4.023)	0.002
UCA1	High/Low	2.693 (1.563–4.641)	< 0.001	1.859 (1.077–3.210)	0.026

### UCA1 depletion suppresses cell proliferation, colony formation, cell migration and invasion and induces G0/G1 cell cycle arrest in HCC cell lines

**Figure 2:**
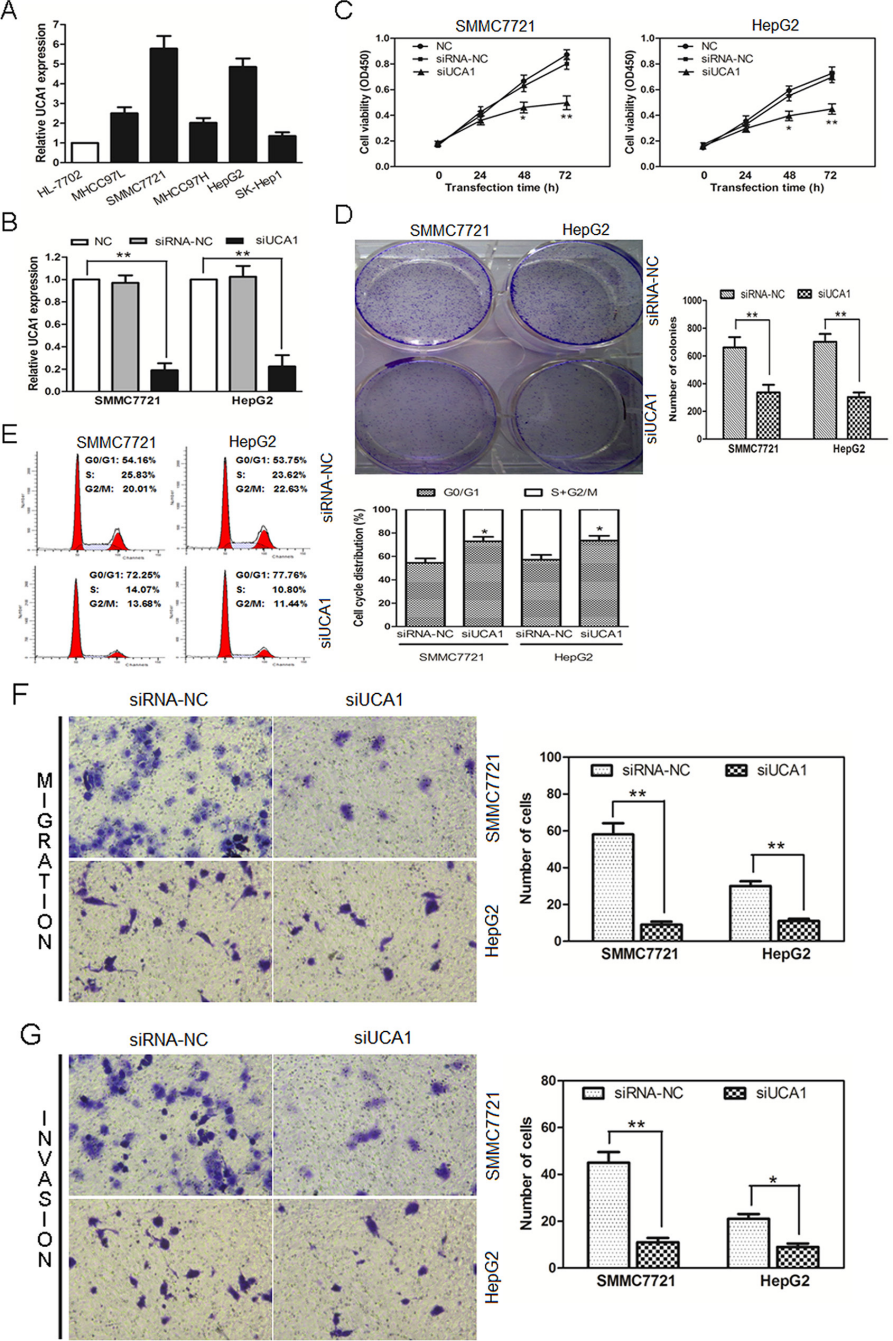
UCA1-knockdown suppresses cell proliferation, colony formation, cell migration, invasion and induces cell cycle arrest of HCC cells **(A)** UCA1 expression levels were analyzed in different liver cell lines by qRT-PCR and GAPDH was treated as internal control. **(B)** UCA1 expression was examined in NC (non-transfected control), siRNA-NC (siRNA non-targeting control) and siUCA1 (siRNA-UCA1) transfected SMMC7721 and HepG2 cells by qRT-PCR. GAPDH was used as an internal control. **(C)** Cell growth viability was assayed in NC, siRNA-NC and siUCA1 transfected SMMC7721 and HepG2 cells by CCK-8 at 0 h, 24 h, 48 h and 72 h time point. **(D)** Colony formation assays were performed in NC, siRNA-NC and siUCA1 transfected SMMC7721 and HepG2 cells (left panel, crystal violet staining; right panel, number of colonies from three independent experiments). **(E)** Cell cycle profile was examined by flow cytometry with propidium iodide staining, cell number were counted according to DNA content of G0/G1, S and G2/M phases (left panel). The statistical results were shown on the right panel. Representative images of migration **(F)** and invasion **(G)** of SMMC7721 and HepG2 cells transfected with siUCA1 and siRNA-NC were showed on the left panel (200 × magnification). The number of migrated and invaded cells was measured in the right panel, respectively, mean ± SD, *P < 0.05, **P < 0.01.

Based on above observations, an analysis of UCA1 expression was carried out among 5 different HCC cell lines (MHCC97L, SMMC7721, MHCC97H, HepG2 and SK-Hep1) and a normal liver cell line (HL-7702). We noted that UCA1 was obviously overexpressed in 5 HCC cell lines than that of HL-7702 cells, especially in SMMC7721 and HepG2 cell lines (Figure 2A). Thus, SMMC7721 and HepG2 cell lines were selected as research represents of HCC cells in the following studies.

Then, we constructed siRNA vector targeting UCA1, namely siUCA1. The knockdown efficiency was obtained about 81% in SMMC7721 and 78% in HepG2 cells after being stably transfected with siUCA1 (Figure 2B). To further assess the potential effects of RNAi-mediated UCA1 silencing on cell proliferation, CCK-8 assay was performed 24, 48 and 72 hours after siRNA transfection. Compared with the non-transfected control (NC) and non-targeting control (siRNA-NC) transfected cells, a significant decrease of cell viability was detected in SMMC7721 and HepG2 cells at 48 or 72 h after treatment with siUCA1; whereas, no significant difference was observed in NC and siRNA-NC transfected cells at each time point (Figure 2C). To further testify the anti-proliferative effect of siUCA1 on the growth of HCC cells, colony formation assay was performed. As shown in Figure 2D, the colony numbers of SMMC7721 and HepG2 cells transfected with siUCA1 were significantly lower than those transfected with siRNA-NC. Thus, the results of colony formation assay were consistent with those of CCK-8 assay and further indicated that siUCA1 could inhibit *in vitro* proliferation of HCC cells.

We further analyzed cell cycle distribution using flow cytometry in siUCA1 treated SMMC7721 and HepG2 cells (Figure 2E). In comparison with siRNA-NC transfected cells, both siUCA1 transfected cell lines showed cell cycle arrest in G0/G1 phase 48 hours after transfection, characterized by the presence of nearly 75% of cells in the G1 phase of the cell cycle, the presence of about 25% of cells in the S+ G2/M phase. The results showed that the G1-S cell cycle progression was inhibited following the silencing of UCA1 in these two HCC cell lines.

To examine the effect of siUCA1 on cell migration, siUCA1 and siRNA-NC transfected SMMC7721 and HepG2 cells were cultured on Transwell apparatus. After 12 h incubation, the percentage of migrated cells in both siUCA1 transfected SMMC7721 and HepG2 cells was significantly less than that in the siRNA-NC transfected cells (Figure 2F). By using a Boyden chamber coated with matrigel, we then determined the effect of siUCA1 on cell invasion after 18 h incubation. Compared with the siRNA-NC transfected cells, both siUCA1 transfected SMMC7721 and HepG2 cells showed obvious decrease in cell invasion (Figure 2G). These data indicates that UCA1 has oncogenic properties with the promotion of metastasis, and siUCA1 can inhibit a migratory and invasive phenotype in HCC cells.

### Knockdown of UCA1 inhibits tumor growth *in vivo*

**Figure 3:**
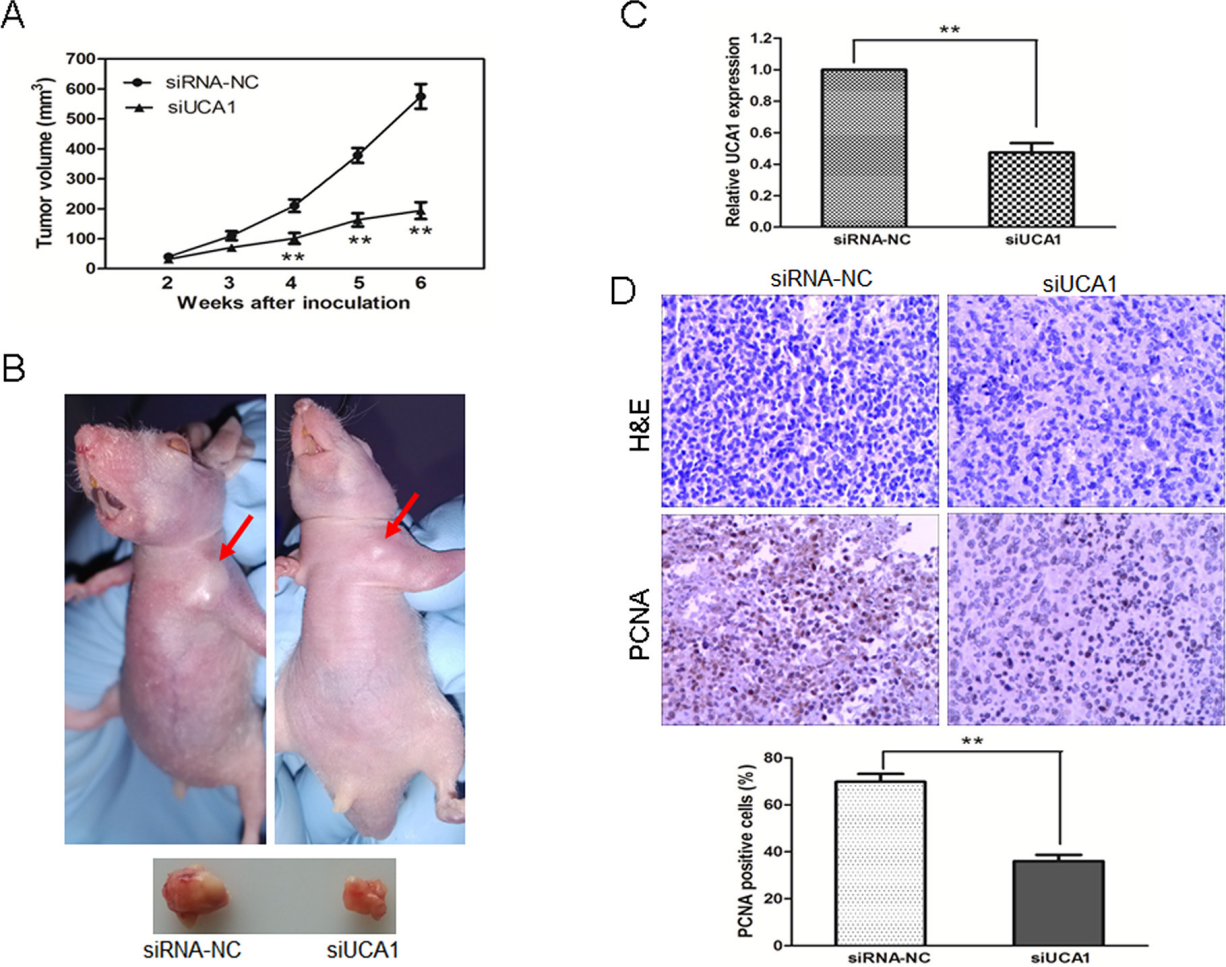
UCA1 depletion inhibits tumor growth *in vivo* **(A)** Tumor growth curves measured after injection of SMMC7721 cells stably transfected with siRNA-NC or siUCA1. The tumor volume was calculated every 7 days from 2 to 6 weeks. **(B)** Photographs of representative tumor formation in nude mice and tumor xenografts 6 weeks after inoculation. **(C)** qRT-PCR analysis of UCA1 expression in tissues of resected tumors formed from siUCA1 or siRNA-NC transfencted SMMC7721 cells. **(D)** Tumors developed from siUCA1 transfected cells showed a lower level of PCNA protein expression than tumors developed from siRNA-NC transfected cells. Upper: H&E staining; Lower: immunostaining (×200). Quantification of immunohistochemical assay was represented as percentage of PCNA positively-stained cells from 5 arbitrarily selected fields, mean ± SD, **P < 0.01.

To assess the effects of siUCA1 on the *in vivo* growth of HCC cells, we applied a xenograft model in which the SMCC7721 cells treated with siUCA1 or siRNA-NC were subcutaneously injected into the flanks of athymic mice and were allowed to develop measurable tumors. There was no animal death in the course of the treatment and no other complications such as skin necrosis were detected due to infection. The tumor formation rate in siRNA-NC transfected group was 90% (9/10); whereas only 60% (6/10) nude mice in siUCA1 transfected group gave rise to tumors. During the whole tumor growth period, tumors from the siUCA1 transfected SMMC7721 cells grew slower than that of siRNA-NC transfected ones (Figure 3A). After 6-week inoculation, the average weight of tumors developed from siUCA1 transfected SMMC7721 cells (217 ± 17 mg) was obviously smaller than those of control mice (592 ± 32 mg) (Figure 3B). Next, qRT-PCR analysis of UCA1 expression and immunostaining analysis of proliferating cell nuclear antigen (PCNA) protein expression were performed in resected tumor tissues. As shown in Figure 3C, the level of UCA1 expression in tumors formed from siUCA1 transfected SMMC7721 cells was significantly lower than that in tumors formed from control cells. In comparison with that in tumors formed from control cells, the positive rate of PCNA expression in tumors developed from siUCA1 transfected SMMC7721 cells was significantly decreased (Figure 3D). These results suggest that UCA1 depletion can inhibit proliferation capacity of HCC cells *in vivo*.

### UCA1 reduces miR-216b expression in HCC

Recently, mounting evidence has showed that lncRNAs contain motif with sequence complementary to miRNAs and have an inhibition effect on miRNAs expression and activity [23–25]. To examine whether UCA1 has a similar mechanism in HCC, prediction of miRNA target sites was performed by the online software Diana Tools. UCA1 RNA contains many elements complementary to various miRNAs seed regions. The expression levels of seven randomly chosen miRNAs were measured in siUCA1 treated SMMC7721 and HepG2 cells by qRT-PCR. Surprisingly, the expression levels of almost all the miRNAs were not or slightly changed (< 1.5-fold) with the exception of miR-216b. In comparison with those of siRNA-NC treatment groups, miR-216b expressions showed *a* > 2-fold increase both in siUCA1 transfected SMMC7721 and HepG2 cells (Supplementary Figure S1A–S1C). These results indicate that UCA1 may play a role in deregulation of miR-216b.

**Figure 4:**
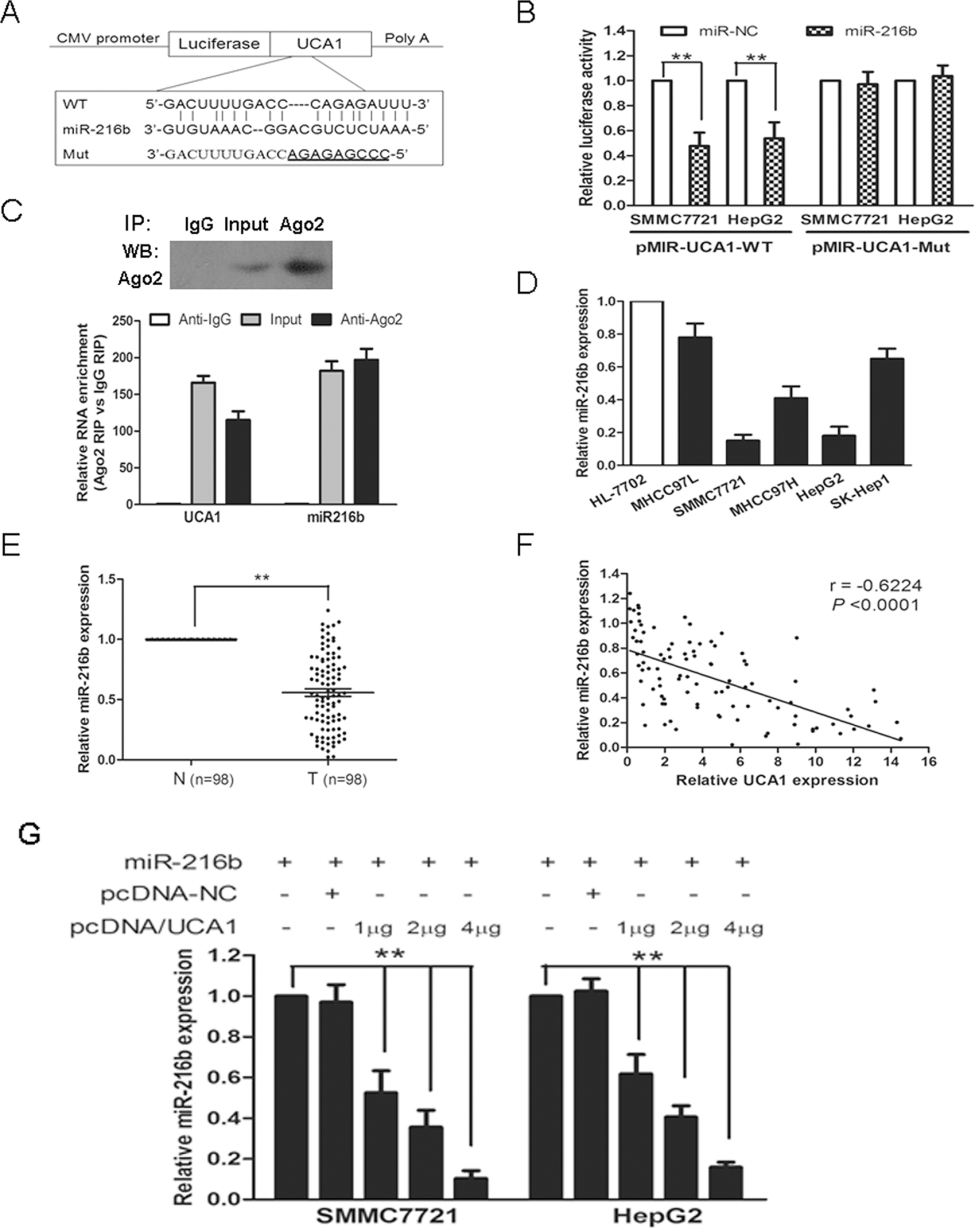
UCA1 reduces miR-216b expression in HCC **(A)** Putative miR-216b-binding sequence of UCA1 RNA. Mutation was generated on the UCA1 RNA sequence in the complementary site for the seed region of miR-216b, as shown. A human UCA1 RNA containing wild type or mutant miR-216b-binding sequence was cloned into pMIR luciferase reporter. **(B)** The wild type (pMIR-UCA1-WT) and mutant (pMIR-UCA1-Mut) reporter plasmids were co-transfected into SMMC-7721 and HepG2 cells with miR-216b or negative control (miR-NC). The normalized luciferase activity in the control group was set as relative luciferase activity. **(C)** Cellular lysates from SMMC-7721 cells were used for RNA immunoprecipitation (RIP) with Ago2 antibody. Detection of Ago2 using IP-western (upper panel), and detection of UCA1 and miR-216b using qRT-PCR. RNA levels were presented as fold enrichment in Ago2 relative to IgG immunoprecipitates (lower panel). **(D)** MiR-216b expression levels were analyzed in different liver cell lines by qRT-PCR and U6 was treated as internal control. **(E)** MiR-216b expression was examined by qRT-PCR and normalized to U6 expression in 98 pairs of HCC tissues (T) compared with corresponding nontumourous liver specimens (N). **(F)** The correlation analysis was performed between UCA1 expression levels and miR-216b expression levels in HCC tissues (*n* = 98). **(G)** miR-216b (1 μg) was co-transfected into SMMC-7721 and HepG2 cells with pcDNA-NC (empty vector, 1 μg) or pcDNA/UCA1 (1 μg, 2 μg, 4 μg). The expression of miR-216b was analyzed by qRT-PCR assay and U6 was used as an internal control. All experiments were at least repeated in triplicate, mean ± SD, **P < 0.01.

Bioinformatics reveal UCA1 RNA contains one conserved target site of miR-216b. To confirm this possibility, the wild type sequence of UCA1 (UCA1-WT) or its mutant sequence (UCA1-Mut) (Figure 4A) was subcloned into the pMIR luciferase reporter and then co-transfected with miR-216b or miR-NC into SMMC-7721 and HepG2 cells. The relative luciferase activity of the pMIR-UCA1-WT was significantly decreased by 52.4% and 47.3% respectively, when miR-216b was co-transfected into SMMC-7721 and HepG2 cells. However, the luciferase activity of pMIR-UCA1-Mut was unaffected in both HCC cell lines by co-transfection with miR-216b (Figure 4B). It is well known that miRNAs may regulate their targets through forming RNA-induced silencing complex (RISC), moreover, recent studies have shown that lncRNAs can act as molecular sponges to regulate the miRNAs activity by associating with RISC [10, 11, 24]. To investigate whether both UCA1 and miR-216b might be in the RISC complex, RNA binding protein immunoprecipitation (RIP) experiments were performed on SMMC7721 cell extracts using antibodies against Ago2, a key component of the RISC complex. We confirmed that the Ago2 antibody precipitated the Ago2 protein from our cellular extract (Figure 4C, upper panel). Moreover, RNA levels of UCA1 and miR-216b in immunoprecipitates were determined by qRT-PCR. As expected, UCA1 and miR-216b were enriched 115-fold and 197-fold, respectively, in Ago2 pellets relative to control IgG immunoprecipitates. (Figure 4C, lower panel). Accordingly, our results suggest that UCA1 is present in Ago2-containing RISC, likely through association with miR-216b, in agreement with our bioinformatic analysis and luciferase assays.

As mentioned above, UCA1 was overexpressed both in HCC tissues and HCC cell lines (Figures 1B, 2A). We then investigated the levels of miR-216b expression in HCC tissues and cell lines. As shown in Figure 4D, the expression of miR-216b was drastically decreased both in SMMC7721 and HepG2 cell lines, which showed an opposite result to UCA1 expression. Moreover, miR-216b expression was also dropped in HCC tissues and an inverse correlation was noted between miR-216b and UCA1 expression levels in HCC tissues (*r* = −0.6224, *P* < 0.0001, Figure 4E, 4F). In consideration of the opposite expression pattern of miR-216b and UCA1 in HCC tissues and cell lines and their co-existing in Ago2-containing RISC, we further analyzed whether UCA1 could antagonize miR-216b expression in HCC. UCA1 RNA was cloned into pcDNA3.1 vector and co-transfected into SMMC7721 and HepG2 cells with miR-216b. As expected, the overexpression of UCA1 resulted in downregulation of miR-216b expression in a dose-dependent manner, whereas, pcDNA-NC exerts no significant inhibitory effect on miR-216b expression both in SMMC7721 and HepG2 cells (Figure 4G). Taken together, our results reveal that UCA1 may act as an endogenous sponge ‘antagomir’ which can reduce miR-216b expression.

### UCA1 reverses the inhibitory effect of miR-216b on the growth and metastasis of HCC cells

**Figure 5:**
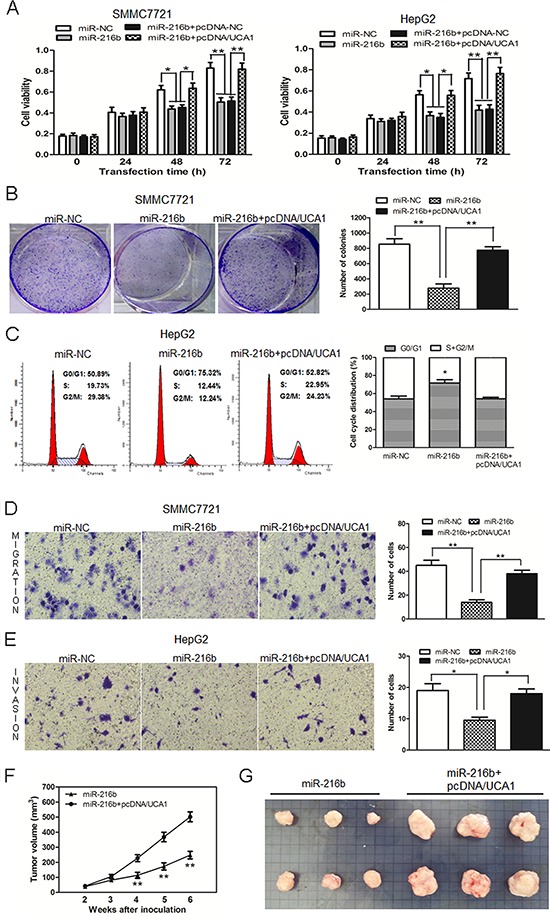
UCA1 reverses the inhibitory effect of miR-216b on cell growth and metastasis of HCC cells *in vitro* and *in vivo* **(A)** Cell growth viability was assayed in miR-NC, miR-216b transfected or miR-216b + pcDNA-NC or miR-216b + pcDNA/UCA1 co-transfected SMMC7721 and HepG2 cells by CCK-8. **(B)** Representative results of colony formation assay after SMMC7721 cells were transfected with miR-NC, miR-216b or co-transfected with miR-216b + pcDNA/UCA1 vectors. **(C)** Cell cycle profile was examined in miR-NC, miR-216b transfected or miR-216b and pcDNA/UCA1 co-transfected HepG2 cells by flow cytometry. Transwell assays were performed to investigate changes in SMMC7721 cell migration **(D)** and HepG2 cell invasiveness **(E)**. Cells were treated with miR-NC, miR-216b or miR-216b + pcDNA/UCA1 vectors. The number of migrated and invaded cells was measured in the right panel, respectively. **(F)** Tumor growth curves measured after injection of SMMC7721 cells stably transfected with miR-216b or co-transfected with miR-216b and pcDNA/UCA1 vectors. The tumor volume was calculated every 7 days from 2 to 6 weeks. **(G)** Photographs of tumor xenografts 6 weeks after inoculation. All experiments were at least repeated in triplicate, mean ± SD, *P < 0.05, **P < 0.01.

In view of the inhibitory effect of UCA1 on miR-216b expression in HCC, we further investigated whether UCA1 had the same effect on the function of miR-216b. Compared with miR-NC treatment groups, miR-216b significantly reduced cell viability in both at 48 and 72 h after transfection into SMMC7721 and HepG2 cells. However, compared with miR-216b or miR-216b + pcDNA-NC treatment groups, the cell viability in miR-216b + pcDNA/UCA1 co-transfected SMMC7721 and HepG2 cells was obviously increased and could basically restore to the original growth activity (Figure 5A). In colony formation assays, the colony numbers of SMMC7721 cells transfected with miR-216b were significantly less than those of miR-NC treated cells. Interestingly, the colony numbers in miR-216b and pcDNA/UCA1 co-transfected SMMC7721 cells were drastically increased than those of miR-216b treated cells, which showed the similar results with those of CCK-8 assay (Figure 5B). The examination of cell cycle profile showed that G0/G1 cell cycle arrest was induced in miR-216b transfected HepG2 cells, inversely, less G0/G1 phase and more S+G2/M phase cells were observed after HepG2 cells were co-transfected with miR-216b and pcDNA/UCA1 vectors (Figure 5C). Our data shows that miR-216b, acting as a tumor suppressor gene, inhibits cell proliferation, colony formation and induces G0/G1 cell cycle arrest, whereas, UCA1 can overturn these inhibitory effects of miR-216b on HCC cells.

On the other hand, in comparison with the miR-NC transfected group, miR-216b could also depress SMMC7721 cell migration and HepG2 cell invasion. Nevertheless, the number of migrated and invaded cells was obviously added both in miR-216b + pcDNA/UCA1 treated two cell lines (Figure 5D, 5E). The results show that UCA1 can mainly regain metastasis potentiality of miR-216b treated HCC cells *in vitro*.

Furthermore, the effects of HCC cells treatment with miR-216b or miR-216b and pcDNA/UCA1 on the *in vivo* growth were also evaluated. During the whole tumor growth period, tumors from the miR-216b and pcDNA/UCA1 co-transfected SMMC7721 cells grew faster than that of miR-216b transfected ones (Figure 5F). After 6-week inoculation, the average weight of tumors developed from miR-216b and pcDNA/UCA1 co-transfected cells (554 ± 26 mg) was obviously larger than those of miR-216b treated group (243 ± 14 mg, Figure 5G), suggesting that UCA1 can invert the inhibition effect of miR-216b on the growth of HCC cells *in vivo*.

### Relationship between UCA1 and the miR-216b mRNA target, FGFR1

**Figure 6:**
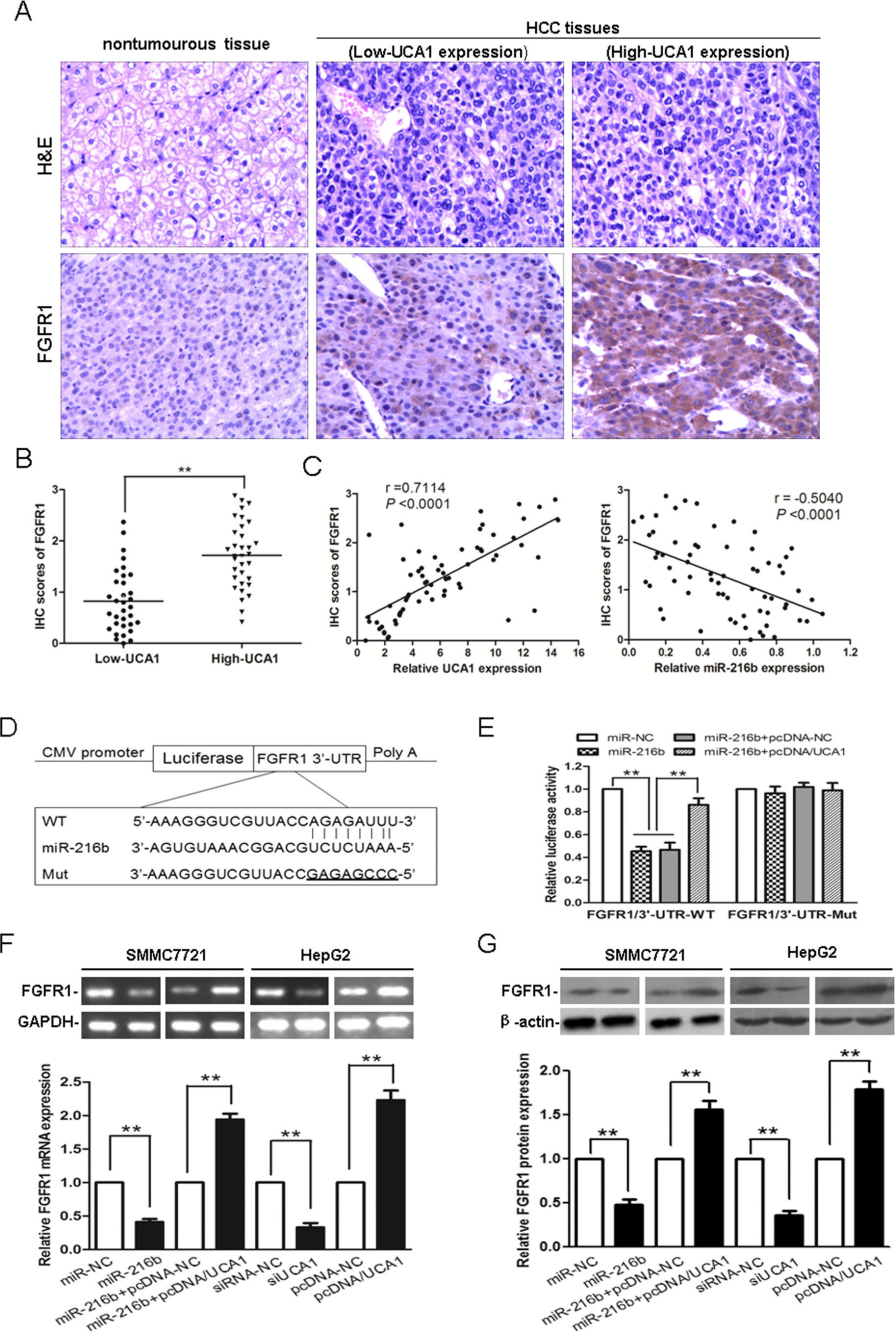
Experimental verification the relationship between UCA1 and the miR-216b mRNA target, FGFR1 **(A)** Representative immunohistochemical staining for FGFR1 from HCC tissues and corresponding nontumourous tissue. Upper: H&E staining; Lower: immunostaining (×200). **(B)** The statistical graph showed that immunohistochemistry (IHC) scores of FGFR1 in high-UCA1 HCC tissues were significantly higher than that of low-UCA1 HCC tissues. **(C)** The correlation analyses were performed between IHC scores of FGFR1 and the levels of UCA1 expression (left panel) or miR-216b expression (right panel) in HCC tissues. **(D)** Putative miR-216b-binding 3′UTR sequence of FGFR1 mRNA. Mutation was generated on the FGFR1 mRNA 3′UTR sequence in the complementary site for the seed region of miR-216b. The wild type or mutant miR-216b-binding FGFR1 mRNA 3′UTR sequence was cloned into pMIR luciferase reporter. **(E)** The wild type (FGFR1/3′-UTR-WT) and mutant (FGFR1/3′-UTR-Mut) pMIR luciferase reporter were co-transfected into SMMC-7721 cells with miR-NC, miR-216b, miR-216b and pcDNA-NC or miR-216b and pcDNA/UCA1. The normalized luciferase activity in the control group was set as relative luciferase activity. qRT-PCR **(F)** and western blotting analyses **(G)** of the levels of FGFR1 mRNA and protein expression following treatment of SMMC-7721 cells with miR-NC, miR-216b, miR-216b + pcDNA-NC or miR-216b + pcDNA/UCA1, and HepG2 cells with siRNA-NC, siUCA1, pcDNA-NC or pcDNA/UCA1, GAPDH and β-actin were used as controls, respectively. The results were reproducible in three independent experiments, mean ± SD, **P < 0.01.

As described above, UCA1 can inhibit miR-216b expression and function in HCC cells. We hypothesized that reduction of miR-216b might decrease repression to its mRNA targets, thereby further facilitated the malignant progression of HCC. Consequently, by performing a computational screen for genes with complementary sites of miR-216b in their 3′-UTR using online softwares including TargetScan (www.targetscan.org), miRanda (www.microrna.org) and miRBase (www.mirbase.org), we found that fibroblast growth factor receptor 1 (FGFR1) was a putative target of miR-216b. Mounting evidence has been reported that FGFR mediates FGF signaling in carcinogenesis and its expression is always dysregulated in many tumor tissues [26, 27]. We then detected the protein expression of FGFR1 by immunohistochemical staining in patients with HCC. As shown in Figure 6A, the immunostaining intensity of FGFR1 in HCC tissues was obviously higher than that of adjacent nontumourous tissues. Moreover, we analyzed FGFR1 protein expression in HCC tissues with different UCA1 levels. The low versus high UCA1 expression was defined as the median value of UCA1 level according to the cohort of tested patients. The levels of FGFR1 protein expression in high-UCA1 HCC tissues were drastically higher than that of low-UCA1 HCC tissues, *P* < 0.01 (Figure 6B). Meanwhile, a significant positive correlation was found between FGFR1 protein expression levels and UCA1 expression levels in HCC tissues (*r* = 0.7114, *P* < 0.0001); whereas, a significant negative correlation was found between FGFR1 protein expression levels and miR-216b expression levels in HCC tissues (*r* = −0.5040, *P* < 0.0001; Figure 6C). In addition, the expression of FGFR1 mRNA was also increased in HCC tissues than that in corresponding nontumourous tissues and an inverse correlation was found between FGFR1 mRNA and miR-216b expression levels in HCC tissues (*r* = −0.7094, *P* < 0.0001). On the other hand, a positive correlation between FGFR1 mRNA and UCA1 expression levels in HCC tissues was also noted (*r* = 0.6116, *P* < 0.0001, Supplementary Figure S2A–S2C). These data indicates that FGFR1 is co-expressed with UCA1 in majority HCC tissues and the interaction of UCA1, miR-216b and FGFR1 might be biologically significant in HCC.

Our luciferase assays were then used to evaluate the possibility of bioinformatical prediction. The wild type 3′-UTR sequence of FGFR1 (3′-UTR-WT) or its mutant sequence (3′-UTR-Mut) (Figure 6D) was subcloned into the pMIR luciferase reporter and then co-transfected with miR-NC, miR-216b, miR-216b + pcDNA-NC or miR-216b + pcDNA/UCA1 into SMMC-7721 cells. As compared with miR-NC treatment group, the relative luciferase activity of the FGFR1/3′-UTR-WT was significantly decreased by 54.7% after co-transfected with miR-216b into SMMC-7721 cells, confirming that FGFR1 was a target of miR-216b. Interestingly, when pMIR-FGFR1/3′-UTR-WT was co-transfected together with miR-216b and pcDNA/UCA1 into SMMC-7721 cells, owing to the presence of UCA1, the luciferase activity of FGFR1/3′-UTR-WT was partly restored, as compared with the miR-216b and miR-216b + pcDNA-NC treatment groups, *P* < 0.01. Moreover, the luciferase activity of FGFR1/3′-UTR-Mut was unaffected in SMMC-7721 cells after co-transfection with any vector (Figure 6E). Our results further confirm that UCA1 acts as an endogenous sponge by binding miR-216b, thus abolishing the miRNA-induced repressing activity on the FGFR1 3′-UTR.

Then we examined FGFR1 expression in cell lines by qRT-PCR and western blotting. As expected, overexpression of miR-216b in SMMC-7721 cells or knockdown of UCA1 in HepG2 cells triggered a markedly silencing effect on FGFR1 mRNA and protein expression. However, the levels of FGFR1 mRNA and protein expression were regained after miR-216b treated SMMC-7721 cells co-transfected with pcDNA/UCA1, resulted from the inhibition of both expression and activity of miR-216b. Furthermore, FGFR1 mRNA and protein expressions were apparently upregulated in pcDNA/UCA1 transfected HepG2 cells, as compared with pcDNA-NC transfected cells, *P* < 0.01 (Figure 6F, 6G). In addition, we also detected FGFR1 protein expression in tumor tissues from nude mice models. Tumors developed from siUCA1 transfected SMMC7721 cells showed a lower level of FGFR1 protein expression than tumors developed from siRNA-NC transfected cells; whereas, tumors developed from miR-216b + pcDNA/UCA1 transfected SMMC7721 cells showed a higher level of FGFR1 protein expression than tumors developed from miR-216b transfected cells (Supplementary Figure S3A, S3B). Taken together, our data strongly suggests that by binding miR-216b, UCA1 modulates the derepression of FGFR1, thereby imposing an additional FGFR1 expression at post-transcriptional regulation level.

### UCA1 promotes HCC malignant progression through ERK signaling pathway

**Figure 7:**
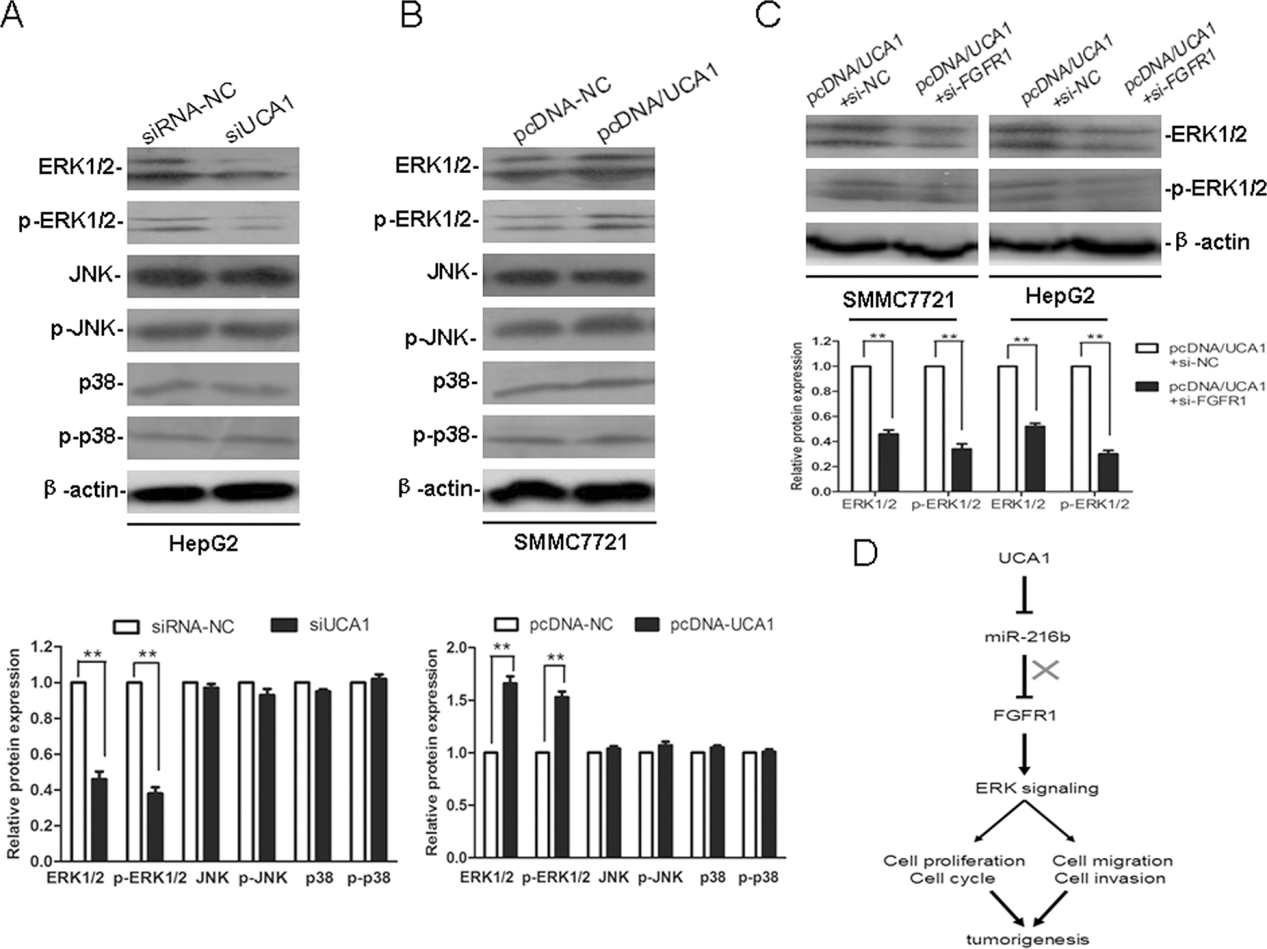
UCA1 promotes HCC malignant progression through ERK signaling pathway Representative western blotting results for ERK1/2, p-ERK1/2, JNK, p-JNK, p38 and p-p38 protein expression from siRNA-NC or siUCA1 treated HepG2 cells **(A)** and pcDNA-NC or pcDNA/UCA1 treated SMMC7721 cells **(B)** (upper panel). **(C)** Representative western blotting results for ERK1/2 and p-ERK1/2 protein expression from pcDNA/UCA1 + si-NC or pcDNA/UCA1 + si-FGFR1 treated HepG2 and SMMC7721 cells. The relative protein expression levels were obtained from three independent experiments, β-actin was used as a control, mean ± SD, **P < 0.01, (lower panel). **(D)** Diagram depicting the regulation mechanism of UCA1 in the tumorigenesis of HCC.

Documents have reported that FGFR mediates FGF signaling, playing crucial roles in cancer cell proliferation, migration, angiogenesis and survival, mainly through activation of the mitogen activated protein kinase (MAPK) signaling pathway, including extracellular signal-regulated kinase (ERK), p38 MAPK and c-Jun N-terminal kinase (JNK) pathways [28, 29]. To further explore the potential mechanism that might be involved in the UCA1-associated malignant progression of HCC, we examined the protein expression levels of ERK1/2, p-ERK1/2, JNK, p-JNK, p38 and p-p38 in HCC cell lines. Western blot analysis revealed that compared with the siRNA-NC, the protein expression levels of ERK1/2 and p-ERK1/2 were markedly reduced in the siUCA1 transfected HepG2 cells. Moreover, compared with the pcDNA-NC, the protein expression levels of ERK1/2 and p-ERK1/2 were notably elevated in the pcDNA/UCA1 transfected SMMC7721 cells. However, the protein expression levels of JNK, p-JNK, p38 and p-p38 were not significantly changed in both siUCA1 treated HepG2 cells and pcDNA/UCA1 treated SMMC7721 cells (Figure 7A, 7B). In addition, compared with the pcDNA/UCA1+ si-NC co-transfected groups, the protein expression levels of ERK1/2 and p-ERK1/2 were significantly decreased in pcDNA/UCA1 and si-FGFR1 co-transfected HepG2 and SMMC7721 cells (Figure 7C). Because ERK signaling plays a central role in the carcinogenesis and maintenance of common cancers, the dysregulated expression of ERK1/2 and p-ERK1/2 also affects the expression of its potential downstream targets, which are responsible for a wide range of biological processes such as cell proliferation and differentiation, cell cycle and survival, cell migration and invasion, etc. Thus, our present results indicate that UCA1 may facilitate HCC malignant progression partly through FGFR1/ERK, rather than FGFR1/JNK or FGFR1/p38 MAPK, signaling pathway.

## DISCUSSION

LncRNAs are transcribed RNA molecules > 200 nucleotides in length that lack significant protein-coding potential, however, they can regulate protein-coding genes at epigenetic, transcriptional, and post-transcriptional levels and play central roles in physiological processes. Increasing evidence suggests that a variety of lncRNAs are frequently aberrantly expressed in cancers, exhibiting spatially and temporally regulated expression patterns. These differential expression lncRNAs are closely related to tumorigenesis, metastasis, prognosis or diagnosis, serving as roles of oncogenes or and tumor suppressor genes [9, 30]. Thus, more efforts should be made to deeply clarify the biological and molecular mechanisms of lncRNAs in cancer.

In this study, we detected the expression of a novel long noncoding RNA UCA1 in HCC samples and their corresponding nontumorous tissues. The results demonstrated that UCA1 was upregulated in HCC tissues and was closely correlated with advanced TNM stage, metastasis and poorer overall survival. Moreover, UCA1 might act as an independent prognostic factor for survival in HCC patients (Figure 1; Tables 1, 2). However, a previous report showed that the elevation of UCA1 in liver cancer (2 in 14 cases) was not as high as in our present study [31]. The probable reasons were the limited number of cases, the highly heterogeneous characteristic of HCC and different primers design. In addition, we identified the function of UCA1 in HCC cells by applying loss-of-function approaches. UCA1 depletion could suppress cell proliferation, colony formation, migration and invasion and induce G0/G1 cell cycle arrest in HCC cell lines *in vitro*, as well as inhibit tumor growth *in vivo* (Figures 2, 3). This is the first report to demonstrate the functional significance of UCA1 expression in human HCC, and our findings indicate that UCA1 functions as an oncogene and promotes HCC malignant progression. Thus, UCA1 holds great promise as a novel diagnostic and prognostic marker and therapeutic target for HCC.

Although the majority of lncRNAs have been shown to play important biological roles and are deregulated in many human cancers, the precise molecular mechanisms by which lncRNAs modulate tumor growth remain largely unknown. In contrast to the smaller non-coding miRNAs, which have been found to be key players in human carcinogenesis, the regulatory mechanisms governing lncRNAs are more diverse and complicated. Besides various functions such as chromatin modification, RNA processing, structural scaffolds, reprogramming of induced pluripotent stem cells and modulation of apoptosis and invasion, etc., intriguingly, lncRNAs have recently been found acting as miRNA sponges or miRNA inhibitors (antagomirs), which interact with miRNAs and modulate the expression of miRNA target genes [10–12]. For example, a cardiac hypertrophy-associated lncRNA, CHRF, acts as an endogenous sponge of miR-489, which can directly bind to miR-489 and regulate its target gene, Myd88, expression and hypertrophy [32]. Moreover, lncRNA HOTAIR may act as a ceRNA, which effectively inhibits the expression of miR-331-3p, thereby modulating the derepression of HER2, a target gene of miR-331-3p, in gastric cancer [33]. In addition, the expression of lncRNA HULC is involved in an auto-regulatory loop in which it's inhibitory to expression and activity of miR-372 allows HULC upregulated expression in liver cancer [34]. There may be some mechanisms that lncRNAs can degrade the binding miRNAs, which is similar to the function of miRNA sponges or antagomirs that promote miRNAs degradation; however, the exact mechanism is still unclear [32]. In this work, we provided further evidence of lncRNA as a miRNA sponge, linking mRNA post-transcriptional regulation network in HCC pathogenesis.

Owing to miR-216b expression, rather than other 6 predicted miRNAs, having a significant increase in siUCA1 treated HCC cells (Supplementary Figure S1), and an inverse correlation being further observed between UCA1 expression and miR-216b expression in HCC tissues and cell lines (Figure 4D–4F), we subsequently supposed that UCA1 might interact with miR-216b, serving as a potent natural miRNA sponge. As expected, a direct binding ability of the miR-216b response elements on the full-length UCA1 RNA was confirmed by luciferase assays (Figure 4B). It was found that UCA1 was mainly localized in cytoplasm which could interact with phosphorylated heterogeneous nuclear ribonucleoprotein I (hnRNP I) by RIP assay, thereby promoting breast tumor growth by competitive suppression of p27 protein level [14]. In this study, RIP experiments provided further support of the positive association between UCA1 and miR-216b in Ago2-containing RISC (Figure 4C). In addition, UCA1 overexpression resulted in down-regulation of miR-216b expression with a dose-dependent manner in HCC cells, suggesting that UCA1, which acted as an endogenous antagomir, could reduce miR-216b expression (Figure 4G). Meanwhile, we further investigated whether UCA1 had the same inhibitory effect on the function of miR-216b. Ectopic overexpression of miR-216b could suppress cell proliferation, colony formation, migration and invasion and induce G0/G1 cell cycle arrest in HCC cell lines *in vitro*, as well as inhibit tumor growth *in vivo*; whereas, upregulation of UCA1 expression could reverse the inhibitory effect of miR-216b on the growth and metastasis of HCC cells *in vitro* and *in vivo* (Figure 5). Taken together, these data is consistent with our hypothesis and previous reports [10–12, 32–34], indicating that UCA1 may serve as an endogenous sponge, inhibiting both miR-216b expression and the function of miR-216b.

To investigate whether UCA1-induced reduction of miR-216b was resulting in a derepression to its mRNA targets and facilitating the malignant progression of HCC, we particularly focused on the miR-216b target gene FGFR1 for further studies. FGFR1 is a member of the fibroblast growth receptor family, which includes FGFR1-4 that serve as receptor tyrosine kinases. The interaction of FGFR1 with high-affinity FGF ligand leads to activation of an intracellular signaling network [26, 27]. Elevated levels of FGFR1 have been found in a number of human cancers, including prostate cancer, lung cancer, gastric cancer, and so on [35–37]. In addition, FGFR1 activation promotes the epithelial-mesenchymal transition (EMT) in several human cancers [38, 39]. Here, we showed that FGFR1 expression could also be regulated by miRNA and lncRNA. We observed that there was a positive correlation between the levels of FGFR1 mRNA or protein expression and the levels of UCA1 expression in HCC tissues. An inverse correlation between the levels of FGFR1 mRNA or protein expression and the levels of miR-216b expression was also noted (Figure 6A–6C; Supplementary Figure S2). Then, luciferase assays confirmed that FGFR1 was a direct target of miR-216b, and UCA1 could abolish the miR-216b-induced repressing activity on the FGFR1 3′-UTR, as well as mRNA and protein expression of FGFR1 (Figure 6E–6G). These data indicates that by binding miR-216b, UCA1 modulates the derepression of FGFR1, thereby imposing an additional FGFR1 expression at post-transcriptional regulation level. Accumulating evidence suggests that FGFR1 cascade plays a crucial role in tumor cell proliferation, angiogenesis, migration and survival. Moreover, FGFR1 inhibition can reduce proliferation and induce cell death in a variety of *in vitro* and *in vivo* tumor models harboring FGFR aberrations, and a growing number of research groups have selected FGFR1 as target for anticancer drug development [29, 40, 41]. Thus, FGFR1 may be co-expressed with UCA1 in majority HCC tissues and the interaction of UCA1, miR-216b and FGFR1 might be biologically significant in the tumorigenesis-regulating network of HCC.

In addition, our western blot analyses further revealed that UCA1 could facilitate HCC malignant progression through FGFR1/ERK signaling pathway (Figure 7A–7C). The binding of FGF-FGFR1 triggers FGFR substrate 2 (FRS2) phosphorylation by FGFR kinase which serves as docking sites for proteins such as son of sevenless (SOS), growth factor receptor-bound protein 2 (GRB2) and GRB2-associated binder-1 (GAB1), allowing assembly of signaling complexes that promote activation of Ras-RAF-mitogen activated protein kinase kinase (MEK)-ERK signaling pathway [28, 29]. Mounting evidence shows that FGFR1/ERK cascade plays a crucial role in the carcinogenesis and maintenance of common cancers, and its dysregulation affects the expression of potential downstream targets and crosstalk with other signaling pathways, which are responsible for a wide range of biological processes such as cell proliferation and differentiation, cell cycle and survival, angiogenesis, cell migration and invasion, and so on. Moreover, the important roles of FGFR1/ERK signal in a variety of cancers make it a potential therapeutic target for cancer therapy [42, 43]. In this study, we further elucidate a novel UCA1-miR-216b-FGFR1-ERK signaling pathway regulatory network, that is UCA1 acting as an endogenous sponge to reduce miR-216b expression, resulting in derepression of FGFR1 expression and activation of FGFR1/ERK signaling pathway in HCC (Figure 7D). Therefore, the findings provide a new clue for understanding the pathogenesis of HCC and provide an intriguing approach for the diagnosis and treatment of HCC.

In summary, our present work highlights that UCA1 acts as an oncogene by promoting malignant progression of human HCC, notably, mechanistic analysis reveals a novel UCA1-miR-216b-FGFR1-ERK signaling pathway regulatory network in HCC. Nevertheless, studies have shown that there are lncRNA transcriptional auto-regulatory loops which may be feedback to control the expression of lncRNA [34, 44]. ERK signaling may activate its down-streaming transcription factors in the nucleus. Further studies are required to address whether these transcription factors have binding sites within the UCA1 gene promoter region, thereby regulating the expression of UCA1. Moreover, UCA1 may potentially regulate a handful of miRNAs while one miRNA can control multiple target genes, and several cross-talk signaling pathways are also involved in this regulatory network in HCC. In addition, only a small number of functional lncRNAs have been well characterized to date in HCC. Therefore, more efforts are needed to better elucidate the function and critical mechanisms of liver-specific lncRNAs in the progression of liver disease, which may undoubtedly enhance our understanding the occurrence and development of HCC and ultimately facilitate the development of lncRNA-directed diagnosis and therapy for this deadly disease.

## MATERIALS AND METHODS

### Tissue samples

A total of 98 patients who were diagnosed as HCC and had undergone routine hepatic resection in the Nanjing First Hospital of Nanjing Medical University and the Third People's Hospital of Nantong from 2009 to 2010 were included in this study. None of the patients had received preoperative radiotherapy or chemotherapy prior to surgical resection. The histological diagnosis and differentiation were evaluated independently by two pathologists according to the WHO classification system. The clinicopathological features are shown in Table 1. Tumor and corresponding non-tumor fresh specimens were snap-frozen in liquid nitrogen and stored at −80°C immediately after resection for the extraction of RNA and protein. The project protocol was approved by the Institutional Review Board of Jiangsu Province Medical Association. All patients provided written informed consent for the use of the tumor tissues for clinical research.

### Cell culture

Five human liver cancer cell lines (MHCC97L, SMMC7721, MHCC97H, HepG2 and SK-Hep1) and a normal liver cell line (HL-7702) were purchased from the Institute of Biochemistry and Cell Biology of the Chinese Academy of Sciences (Shanghai, China). Cells were maintained in Dulbecco's modified Eagle's medium (DMEM) with 10% fetal bovine serum (Thermo Scientific HyClone, Beijing, China), 100 U/ml penicillin and 100 mg/ml streptomycin in humidified air at 37°C with 5% CO2.

### Real-time PCR

Total RNA was extracted by Trizol reagent (Invitrogen, Carlsbad, CA, USA). Reaction mixture (20 μl) containing 1 μg of total RNA was reversely transcribed to cDNA by using PrimeScript RT-polymerase (Takara, Dalian, China). Quantitative PCR was performed on the cDNA using specific primers (Sangon, Shanghai, China) for UCA1 and FGFR1. GAPDH was used as an internal control. Specifically, stem-loop reverse transcriptase polymerase chain reactions (RT-PCR) for mature miRNAs (miR-216b, miR-665, miR-326, miR-212-5p, miR-338-3p, miR-567, miR-136-3p) were performed. The primers for miRNAs and U6 snRNA were purchased from RiboBio (Guangzhou, China). All reactions were carried out on the Applied Biosystems 7000 Sequence Detection System (Applied Biosystems, Foster City, CA, USA). Relative expression levels were calculated as ratios normalized against those of GAPDH or U6 snRNA. Comparative quantification was determined using the 2^−ΔΔCt^ Method. Primers can be found in the Supplementary Table S1.

### Construction of reporter and recombinant vector

To construct UCA1 small interfering (si)RNA vector, the self-complementary hairpin DNA oligos targeting UCA1 were synthesized, named as siUCA1. A negative control, named as siRNA-NC, was also designed (See in the Supplementary Table S1). DNA oligos were annealed and subcloned into pGCsi/H1/Neo/GFP plasmid vector (Genechem, Shanghai, China). To construct expression vectors, UCA1 cDNA and miR-216b precursors with flanking sequences in both sides were amplified and cloned to pcDNA3.1 in BamhI/XhoI sites (Invitrogen). To construct luciferase reporter vectors, FGFR1 3′-untranslated regions (UTR) and UCA1 cDNA fragment containing the predicted potential miR-216b binding sites or mutant sites were amplified by PCR, and then cloned to pMIR-Report Luciferase vector in MluI/HindIII sites (Ambion, Austin, TX, USA). Site-directed mutagenesis of the miR-216b target sites in the FGFR1 3′-UTR and UCA1 cDNA were performed using the Quick-change mutagenesis kit (Stratagene, Heidelberg, Germany) and named FGFR1/3′-UTR-Mut, pMIR/UCA1-Mut. Primers for subcloning and plasmid construction were listed in Supplementary Table S1.

### Luciferase assay

Cells grown in the 96-well plate were co-transfected with either empty vector or miR-216b and luciferase reporter comprising 3′UTR of FGFR1, wild type or mutant UCA1 fragment, using Lipofectamie 2000 (Invitrogen). Cells were harvested 48 h after transfection and luciferase activity was measured as chemiluminescence in a luminometer (PerkinElmer Life Sciences, Boston, MA, USA) using the Dual-Luciferase reporter assay system (Promega, Madison WI, USA) according to the manufacturer's protocol.

### RNA immunoprecipitation (RIP) assay

RNA immunoprecipitaion used the Magna RIP RNA-Binding Protein Immunoprecipitation Kit (Millipore, Billerica, MA, USA) and the Ago2 (Millipore) antibody according to the manufacturer's protocol. Briefly, cells were lysed in RIP lysis buffer, then 100 μl of whole cell extract was incubated with RIP buffer containing A+G magnetic beads conjugated with human anti-Ago2 antibody, normal mouse IgG (Millipore) as a negative control and Anti-snRNP70 as a positive control (Millipore). Samples were incubated with Proteinase K with shaking to digest the protein and then immunoprecipitated RNA was isolated, then qRT-PCR was performed to detect UCA1 and miR-216b in the precipitates.

### Western blot analysis

The SMMC7721 and HepG2 cells were lysed with denaturing SDS-PAGE sample buffer using standard methods. Protein lysates were separated by 10% SDS-PAGE and transferred onto nitrocellulose membranes. The membranes were blocked with TBS containing 0.1% Triton X-100 and 5% nonfat milk overnight at 4°C, then were incubated with anti-human FGFR1 (Abcam, Cambridge, MA, USA), ERK1/2, p-ERK1/2, JNK, p-JNK, p38 and p-p38 and β-actin antibody (Santa Cruz Biotech, Santa Cruz, CA) at 4°C overnight. After being washed, the membranes were incubated with HRP-conjugated anti-IgG at room temperature for 2 hour. Signal detection was carried out with an ECL system (Amersham Pharmacia, Piscataway, NJ, USA).

### Immunohistochemical staining

The streptavidin-peroxidase (SP) staining technique was used to detect protein following antigen retrieval by microwave treatment. After blocking endogenous peroxidase activity by incubating in 3% H_2_O_2_ for 10 min, specimens were rinsed with PBS, then incubated with FGFR1 and PCNA antibody at 4°C overnight. Specimens were rinsed with PBS and incubated at room temperature for 30 min with secondary antibody, the samples were exposed to streptavidin-peroxidase for another 30 min. After being rinsed with PBS, diaminobenzidine (DAB) solution was used. Counterstaining was performed with hematoxylin. The substitution of PBS for primary antibody was used as negative control. Staining intensity was scored manually by two independent experienced pathologists as no staining = 0, weak staining = 1, moderate staining = 2, and strong staining = 3. Tumor cells in 5–10 fields were randomly selected and scored based on the percentage of positively stained cells (0–100%). The final immunohistochemistry (IHC) score was calculated by multiplying the intensity score with the percentage of positive cells.

### CCK-8 assay cell growth viability

Cells at a concentration of 5 × 10^3^ per well were seeded in the 96-well plate and incubated for 24 h, 48 h, 72 h, respectively. Cell growth viability was measured with a Cell Counting Kit-8 (Beyotime, Shanghai, China), following the manufacturer's instructions. Absorbance (A) was then recorded at 450 nm using Elx800 Reader (Bio-Tek Instruments Inc., Winooski, VT, USA).

### Colony formation assay

The transfected SMMC7721 or HepG2 cells were placed in a fresh six-well plate and maintained in DMEM containing 10% fetal bovine serum. After 24 h, the medium was replaced with new medium containing G418 (400 mg/ml). After 14 days, cells were fixed with methanol and stained with 0.1% crystal violet. Visible colonies were manually counted.

### Migration and invasion assays

For transwell migration assays, SMMC7721 or HepG2 cells transfected cells (4 × 10^5^) were plated in the top chamber with the non-coated membrane (24-well insert; pore size, 8 μm; BD Biosciences, San Jose, CA, USA). For invasion assays, matrigel (BD biosciences) was polymerized in transwell inserts for 45 min at 37°C. In both assays, cells were plated in the top chamber in medium without serum; the lower chamber was filled with 10% FBS and EGF (25 ng/ml) (Sigma, St Louis, MO, USA) was used as a chemoattractant. Cells were incubated for 24 h and the cells that did not migrate or invade through the pores were removed by a cotton swab. Cells on the lower surface of the membrane were stained with crystal violet and counted.

### Cell cycle analysis by flow cytometry

The trypsinized cells (1 × 10^6^) were fixed in 70% ethanol at −20°C for 24 h. The fixed cells were then washed with PBS, and incubated with RNase A (0.25 mg/ml) for 30 min at 37°C, and 5 μl of propidium iodide (KeyGen, Nanjing, China) was then added to the cell suspension. The mixture was incubated at room temperature for 30 min in the dark. The suspended cells were analyzed for cell cycle using the FACS Calibur Flow Cytometer (BD Biosciences, San Jose, CA, USA).

### Animal experiments

Animal experiments were performed with the approval of the Institutional Committee for Animal Research and in conformity with national guidelines for the care and use of laboratory animals. SiUCA1, siRNA-NC, miR-216b transfected or miR-216b and pcDNA/UCA1 co-transfected SMCC7721 cells (1 × 10^7^ cells in 100 μl) were injected subcutaneously into the flanks of each 6-week-old BALB/c athymic nude mice. Tumor growth was examined weekly for at least 6 weeks. Then the mice were killed, necropsies were performed, and tumors were weighted. Tumor volumes were calculated by the following formula: V = πAB^2^/6, where A is the largest diameter, and B is the perpendicular diameter. The tumor tissues were used to perform immuno-staining analysis of PCNA protein expression.

### Statistical analysis

The SPSS15.0 software was used for general statistical. The significance of differences between groups was estimated by Student's *t*-test, one-way analysis of variance (ANOVA), χ2 test or Wilcoxon test, as appropriate. Survival rate were calculated by the Kaplan-Meier method with the log-rank test applied for comparison. Survival data were evaluated using univariate and multivariate Cox proportional hazards model. All tests performed were two sided and the criterion for statistical significance was taken as *P* < 0.05.

## SUPPLEMENTARY FIGURE AND TABLES


