# Supplementary material for: Upregulated lncRNA-UCA1 contributes to progression of hepatocellular carcinoma through inhibition of miR-216b and activation of FGFR1/ERK signaling pathway
Source: Oncotarget. 2015 Feb 11;6(10):7899–917. doi: 10.18632/oncotarget.3219 (PMC4480724; doi:10.18632/oncotarget.3219)
Supplement: Supplementary file 1 [file oncotarget-06-7899-s001.pdf]

SUPPLEMENTARY FIGURES AND TABLE

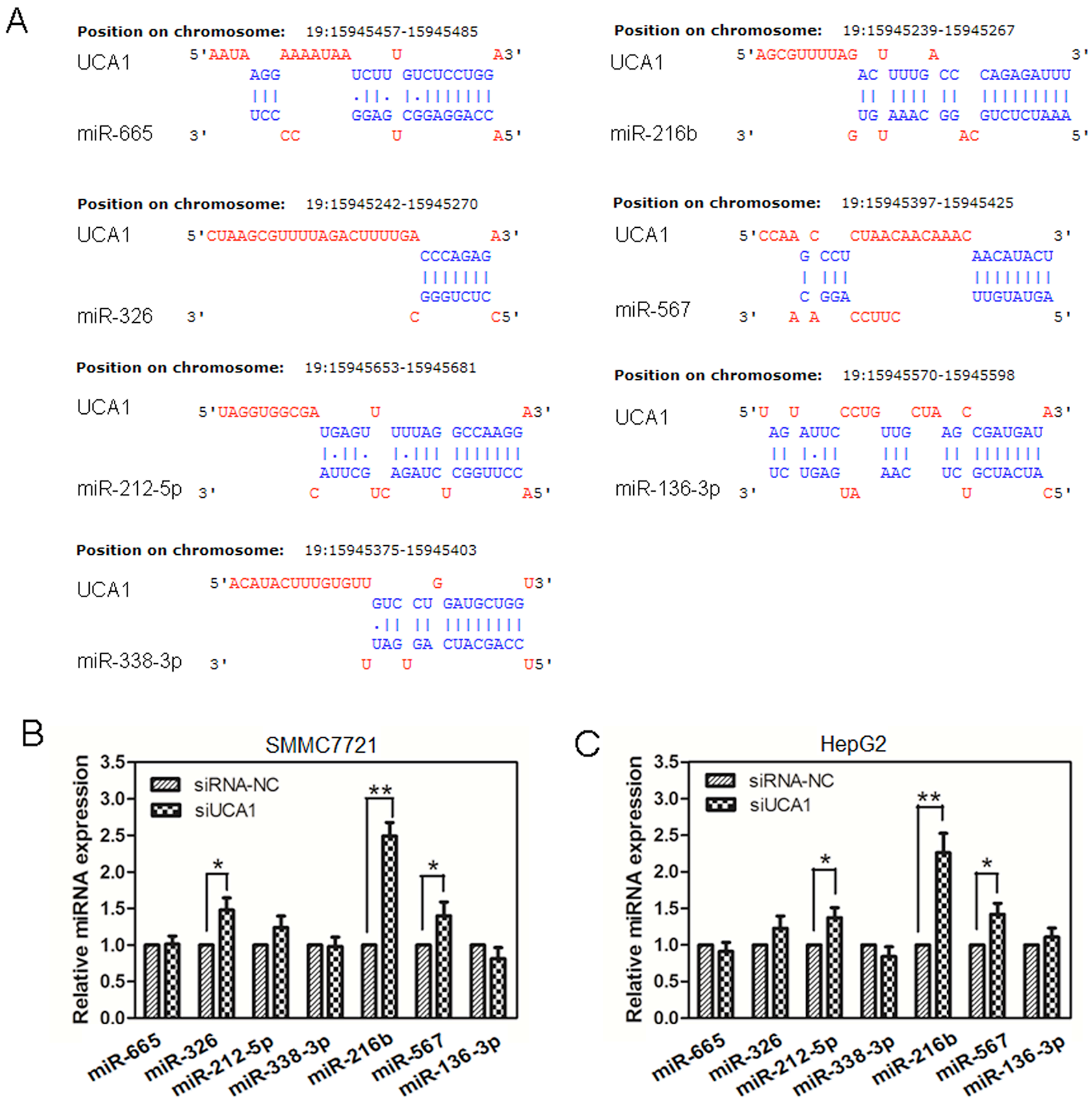

Supplementary Figure S1: MiRNAs expression in siUCA1 transfected HCC cell lines. **(A)** Potential binding sites of seven randomly predicted targeting-miRNAs of UCA1, namely miR-665, miR-326, miR-212-5p, miR-338-3p, miR-216b, miR-567, miR-136-3p, respectively. The expression of miRNAs was detected in siUCA1 transfected SMMC7721 cells **(B)** and HepG2 cells **(C)** by qRT-PCR. All experiments were at least repeated in triplicate, mean±SD, **P* < 0.05, ***P* < 0.01, versus siRNA-NC.

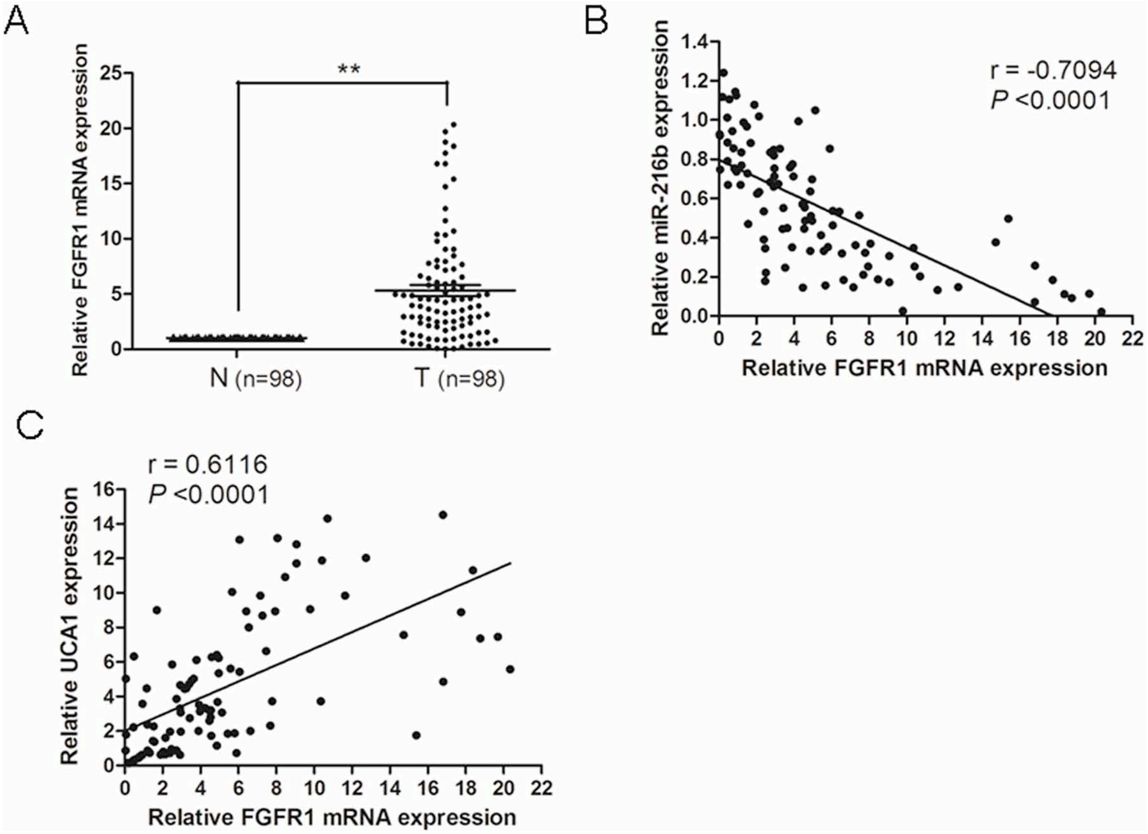

Supplementary Figure S2: Relative FGFR1 mRNA expression in HCC tissues. (A) FGFR1 mRNA was examined by qRT-PCR and normalized to GAPDH expression in 98 pairs of HCC tissues (T) compared with corresponding nontumourous liver specimens (N), $^{**}P < 0.001$. The correlation analyses were performed between the levels of FGFR1 mRNA expression and the levels of UCA1 expression **(B)** or miR-216b expression **(C)** in HCC tissues ($n = 98$).

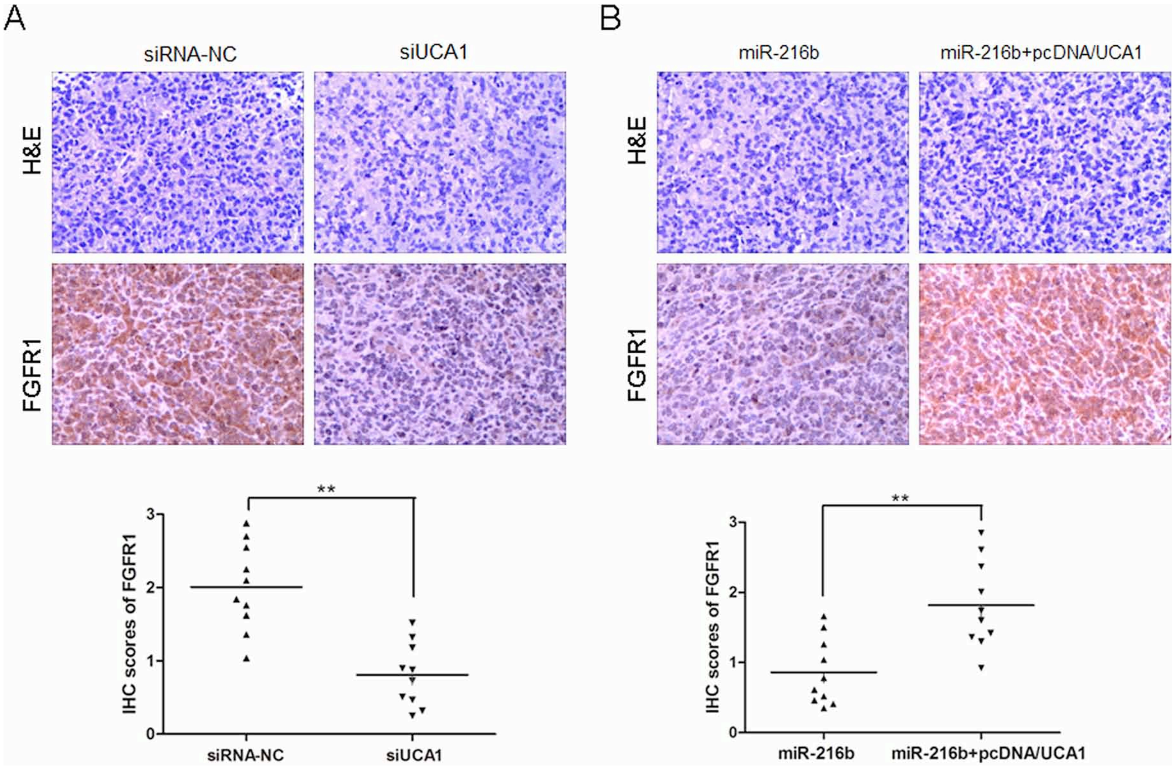

Supplementary Figure S3: Immunohistochemical staining for FGFR1 in tumor tissues from nude mice models. **(A)** Representative tumor tissues developed from siRNA-NC or siUCA1 transfected SMMC7721 cells. **(B)** Representative tumor tissues developed from miR-216b or miR-216b + pcDNA/UCA1 transfected SMMC7721 cells. Upper: H&E staining; Lower: immunostaining (×200). Quantification assays were represented as immunohistochemistry (IHC) scores of FGFR1 from 10 arbitrarily selected fields, $^{**}P < 0.01$.

Supplementary Table S1: Primers and siRNA seguences used in this study

Primers for qRT-PCR	
**Primer name**	**5′-3′**
miR-216b-F	CAGGCACACACTTACCCGTA
miR-216b-R	GCAGGGTCCGAGGTATTC
miR-665-F	ACGCCAACCAGGAGGCTGAG
miR-665-R	GCAGGGTCCGAGGTATTC
miR-326-F	ATTGAACCTCTGGGCCCTTC
miR-326-R	GCAGGGTCCGAGGTATTC
miR-212-5p-F	ATCGCCACCTTGGCTCTAGA
miR-212-5p-R	GCAGGGTCCGAGGTATTC
miR-338-3p-F	ATTGAAGGAGTCGCCTGAGT
miR-338-3p-R	GCAGGGTCCGAGGTATTC
miR-567-F	ACGCCAAGTATGTTCTTCCA
miR-567-R	GCAGGGTCCGAGGTATTC

(*Continued*)

Primers for qRT-PCR	
**Primer name**	**5′-3′**
miR-136-3p-F	ATCGCCCATCATCGTCTCAA
miR-136-3p-R	GCAGGGTCCGAGGTATTC
U6-F	CTCGCTTCGGCAGCACA
U6-R	AACGCTTCACGAATTTGCGT
GAPDH-F	GGTGGTCTCCTCTGACTTCAA
GAPDH-R	GTTGCTGTAGCCAAATTCGTTGT
FGFR1-F	AACCTGACCACAGAATTGGAGGCT
FGFR1-R	ATGCTGCCGTACTCATTCTCCACA
UCA1-F	CTCTCCTATCTCCCTTCACTGA
UCA1-R	CTTTGGGTTGAGGTTCCTGT
**Primers for subcloning and plasmid construction**	
**Primer name**	**5′-3′**
pCDNA/UCA1-F	GGATCCGATCTCTCCTCTTCCTCCTG
pCDNA/UCA1-R	CTCGAGAGGAAGATTTCTTTTCTGTC
pCDNA/pre-miR-216b-F	GGATCCTCCTTCATTCTCTATGGA
pCDNA/pre-miR-216b-R	CTCGAGGTTACAAACAACCTTCTT
pMIR-UCA1-F	ACGCGTGATCTCTCCTCTTCCTCCTG
pMIR-UCA1-R	AAGCTTAGGAAGATTTCTTTTCTGTC
pMIR-FGFR1/3′-UTR-F	CAGTCGACAATCACTCCTGGGAAGATCTCATTG
pMIR-FGFR1/3′-UTR-R	GGTGGATCCAGGGCCACAAGGTGGACAATCGG
**siRNA Oligos**	
**name**	**5′-3′**
siUCA1-sense	GATCCGGCTAATATGCCTGATTACTTTCAAGAG AAGTAATCAGGCATATTAGCTTTTTTGGAAA
siUCA1-antisense	AGCTTTTCCAAAAAAGCTAATATGCCTGATTAC TTCTCTTGAAAGTAATCAGGCATATTAGCCG
siRNA-NC-sense	GATCCGGCCTGTTTAGGTGGTCTTTTCAAGAGA AAGACCACCTAAACAGGCTTTTTTGGAAA
siRNA-NC-antisense	AGCTTTTCCAAAAAAGCCTGTTTAGGTGGTCTT TCTCTTGAAAAGACCACCTAAACAGGCCG
